# Medical Editors’ Association

**Published:** 1915-12

**Authors:** 


### EDITORIAL DEPARTMENT

MRS. F. E. DANIEL, Publisher and Managing Editor.
DR. R. H. L. BIBB, Associate Editor.

CONTRIBUTING EDITORS:

Dr. H. Sheridan Baketel, New York, editor *Medical Times:* Dr. M. H. Boerner, Austin, Texas; Dr. G. Henri Bogart, Paris, Ill.; Dr. M. M. Carrick, Dallas, Texas; Dr. Edward H. Carey, Dean Medical Department, Baylor University, Dallas, Texas; Dr. W. L. Crosthwaite, Waco, Texas; Dr. T. D. Crothers, Hartford, Conn.; Dr. H. W. Cummings, Hearne, Texas; Dr. Oscar Dowling, New Orleans, President Louisiana State Board of Health; Havelock Ellis, M. D., London, Eng.; Dr. May Agnes Hopkins, Dallas, Texas; Dr. A. W. Fly, Galveston, Texas; Dr. G. B. Foscue, Waco, Texas; Dr. E. S. Goodhue, Holualoa, Kona, Hawaii; Dr. M. B. Grace, Seguin, Texas; Dr. Joe Gilbert, Austin, Texas; Dr. Charles H. Hughes, St. Louis, editor *Alienist and Neurologist*; Dr. James G. Kiernan, Chicago; Dr. George H. Lee, Galveston, Texas; Dr. John T. Moore, Houston, Texas; Dr. Frank Paschal, San Antonio, Texas; Dr. John Preston, Austin, Texas, Superintendent State Lunatic Asylum; Dr. G. Wilse Robinson, Kansas City, Mo.; Dr. Bacon Saunders, Fort Worth, Texas; Dr. Allen J. Smith, Philadelphia, Medical Department, University of Pennsylvania; Dr. Z. T. Scott, Austin, Texas; Dr. Ralph Steiner, Austin, Texas, President Texas State Board of Health; Dr. Walter S. Sutton, Kansas City, Mo.; Dr. A. E. Sweatland, Nacogdoches, Texas; Dr. John S. Turner, Dallas, Texas; Dr. Charles Venable, San Antonio, Texas.

### Medical Editors' Association.

Few persons realize that the Medical Editors' Association was organized in the 80's with the late Dr. N. S. Davis as President, and Henry O. Marcy, M. D., of Boston, Mass., as Secretary.

The object of this Association was to combine their editorial interests and energies, and by personal acquaintance, and frequent meetings, settle on some policies that would be mutually helpful.

At first the Association combined about eight or ten leading editors, and the papers offered were personal suggestions, and views concerning the best way of conducting a journal and interesting the medical public.

This society grew in popularity and interest, and finally culminated in a social banquet, after the reading of many papers. Nearly all the leading editors of the country took an active part, and the matter discussed was intensely practical.

The banquet assumed unusual prominence, and was the occasion of most startling comments, criticisms and jokes that were very enjoyable, because of their realism. The leading editors did not hesitate to criticise and laugh down the weakness of their brothers, and if they became angry for this criticism the fun increased, and the good humor that followed was very amusing.

As the years rolled around it was found necessary to have the meeting of this Association the day before the American Medical Association met, and end the meeting with a dinner, to which many prominent men were invited. Instead of being a mutual admiration gathering, it was a kind of clearing house and general airing of personal opinions, criticisms and jokes, that had the good purpose of clearing away the egoisms of many persons.

Of course, someone was offended and a joke at their expense sunk deep the more real and true it was. After ten or twelve months of meditation these hurt persons came back and seemed to take as much interest in the jokes and witticisms played on others.

It was clear to the readers of all medical journals that this Association had a very fine effect on the editorial work. There were less bickerings, less slang and sarcasm, and a higher tone of statement and criticism. The papers read before the Association were constantly growing better, both scientifically and from a literary point of view. Several of them were reprinted and given a very wide circulation, and medical men realized that the editors of journals were equal to any of the specialists in scientific discernment, and skill of expression and recognition of the facts.

Of late years, Dr. Joseph MacDonald, Jr., of New York, Managing Editor of the *American Journal of Surgery*, has been Secretary and Treasurer, and under his genial and skillful management the society has become very prominent, and its meetings and banquets have attracted large numbers of prominent men.

The banquet has been the great social occasion of the A. M. A. meetings, and at no other gathering or dinner was there such witty comments on men and events, and so little praise and mutual admiration heard.

A spirit of humor and jovial fellowship, regardless of the position or prominence of its members, appeared. The editors expressed themselves freely without reserve or fear of offending, laughing at the follies and weaknesses of the men in the profession, high and low, all in the spirit of good humor and frankness. This was expressed in the opinion of a visiting lawyer, who said: "I never heard such genial criticism of men and events as you editors indulge in at these dinners. I think I know more of the profession from hearing what is said at the banquets than from any other source."

Of course, at meetings of this kind, someone was permanently offended, and, in the course of time, a rival society was organized. It was supposed to be limited to the editors of State journals. Their meetings are held probably at the same time, but evidently behind closed doors, and are not open to the public, so the subject of their discussions and work is practically unknown.

In the meantime the old society goes on. The annual meeting was held last month in New York, and was marked by the same group of clear, forcible discussions of journalistic topics, and the election to the presidency of Dr. E. C. Register, Editor of the *Charlotte Medical Journal*. This was a fitting tribute to a very highly cultivated doctor, whose work has been prominent both in the editorial field, and elsewhere, for many years.

The banquet which followed was equal to those held in previous years. The humor was a little more rollicking, classic and sharper than ever, and it was the opinion of old members that the medical editors were becoming more prominent and vigorous, as well as influential in medical literature.

The Secretary publishes a paper in which the annual proceedings and papers appear, and editors who are members make such note of the papers and transactions, as they think will be interesting to their readers.

The fact of most significance is this, that there still exists an independent organization of medical journals who claim the right to use their own judgment in the conduct of their papers, in the articles published, according to their judgment of the needs and necessities of the medical public. This is true democracy, a democracy which science upholds and which is the foundation of all progress and development. T. D. C.

